# Blood Count-Based Inflammatory Indices Do Not Improve Prediction of Acute Kidney Injury in Cardiogenic Shock

**DOI:** 10.14740/jocmr6666

**Published:** 2026-08-26

**Authors:** Jackson Rajendran, Shubhangi Sharma, Franklyn Vega Batista, Anshu Sutihar, Sharon Paul, Song Peng Ang, Saria Qaiser, Cristina Rodriguez, Madison Laezzo, Jose Iglesias

**Affiliations:** a Department of Internal Medicine, Rutgers Health Robert Wood Johnson Community Medical Center, Toms River, NJ 08755, USA; b Department of Medicine, Division of Cardiology, The University of Arizona Sarver Heart Center, Tucson, AZ 85724, USA; c Department of Critical Care Medicine, Rutgers Health Robert Wood Johnson Community Medical Center, Toms River, NJ 08755, USA; d Department of Critical Care Medicine, Prisma Health University of South Carolina, Columbia, SC 29203, USA; e Department of Medicine, Hackensack Meridian School of Medicine, Nutley, NJ 07110, USA; f Department of Nephrology, Rutgers Health Robert Wood Johnson Community Medical Center, Toms River, NJ 08755, USA

**Keywords:** Acute kidney injury, Biomarkers, Cardiogenic shock, Critical care, Risk assessment

## Abstract

**Background:**

Acute kidney injury (AKI) is common in cardiogenic shock and is associated with poor outcomes. Whether routinely available inflammatory indices add useful information for AKI risk stratification in this population remains unclear.

**Methods:**

We performed a retrospective multicenter cohort study using the eICU Collaborative Research Database. Adult intensive care unit (ICU) patients with cardiogenic shock were included if laboratory components required to calculate inflammatory indices were available. All indices were derived from a single set of laboratory values obtained on ICU admission rather than from serial measurements. AKI was defined using creatinine-based Kidney Disease: Improving Global Outcomes criteria and renal replacement therapy status. We evaluated neutrophil to lymphocyte ratio, platelet to lymphocyte ratio, monocyte to lymphocyte ratio, systemic immune-inflammation index, systemic inflammation response index, aggregate index of systemic inflammation, and neutrophil percentage to albumin ratio. Missing covariate data were handled using multiple imputation. Multivariable logistic regression, false discovery rate correction, elastic-net analysis, calibration, and decision-curve analysis were used to assess associations and incremental discrimination.

**Results:**

The final cohort included 419 patients, of whom 273 (65.1%) developed AKI. Baseline creatinine was the strongest variable associated with AKI in the primary multivariable model. Chronic kidney disease was associated with AKI when creatinine and blood urea nitrogen were excluded. Inflammatory indices were not independently associated with AKI after multivariable adjustment and false discovery rate correction. The base clinical model had modest discrimination, which improved after adding renal laboratory variables. Adding inflammatory indices provided little to no meaningful incremental discrimination, and inflammatory indices were not consistently retained in elastic-net models.

**Conclusion:**

In this multicenter ICU cohort of patients with cardiogenic shock, AKI was associated mainly with baseline renal dysfunction and illness severity. Routine inflammatory indices did not provide meaningful incremental discrimination beyond established clinical and renal variables for AKI during ICU hospitalization. These findings apply to blood count-derived indices and do not exclude a contribution of inflammation to AKI in cardiogenic shock.

## Introduction

Cardiogenic shock is associated with substantial morbidity and mortality. Outcomes are determined not only by cardiac dysfunction but also by failure of other organs. Acute kidney injury (AKI) is one of the most common clinically important complications in this setting, and is associated with a worse prognosis [1–3]. In cardiogenic shock, a sudden decrease in cardiac output can cause renal hypoperfusion. Because the kidney is highly sensitive to changes in perfusion and oxygen delivery, this hemodynamic insult can quickly precipitate AKI [4–6].

During the past years, many studies have investigated the prevalence of AKI in cardiogenic shock. This present study focuses on risk factors, including demographic features and comorbid conditions, and evaluates biochemical variables associated with AKI. We also evaluated novel inflammatory indices that are calculated from routine laboratory tests, including the systemic immune-inflammation index (SII), the aggregate index of systemic inflammation (AISI), monocyte to lymphocyte ratio (MLR), neutrophil to lymphocyte ratio (NLR), platelet to lymphocyte ratio (PLR), neutrophil percentage to albumin ratio (NPAR), and systemic inflammation response index (SIRI) [7]. These indices may reflect activation of the systemic immune response and illness severity and may be useful for assessing the prognosis of patients who have undergone percutaneous coronary intervention (PCI) [8]. The inflammatory cascade in AKI may begin when tubular epithelial cells and vascular endothelial cells respond to ischemia-reperfusion or nephrotoxins by releasing damage-associated molecular patterns [9]. We therefore sought to determine whether these markers of systemic immune activation were independently associated with AKI in critically ill patients with cardiogenic shock, and whether they added useful discriminatory value for early risk stratification.

## Methods

**Figure 1:**
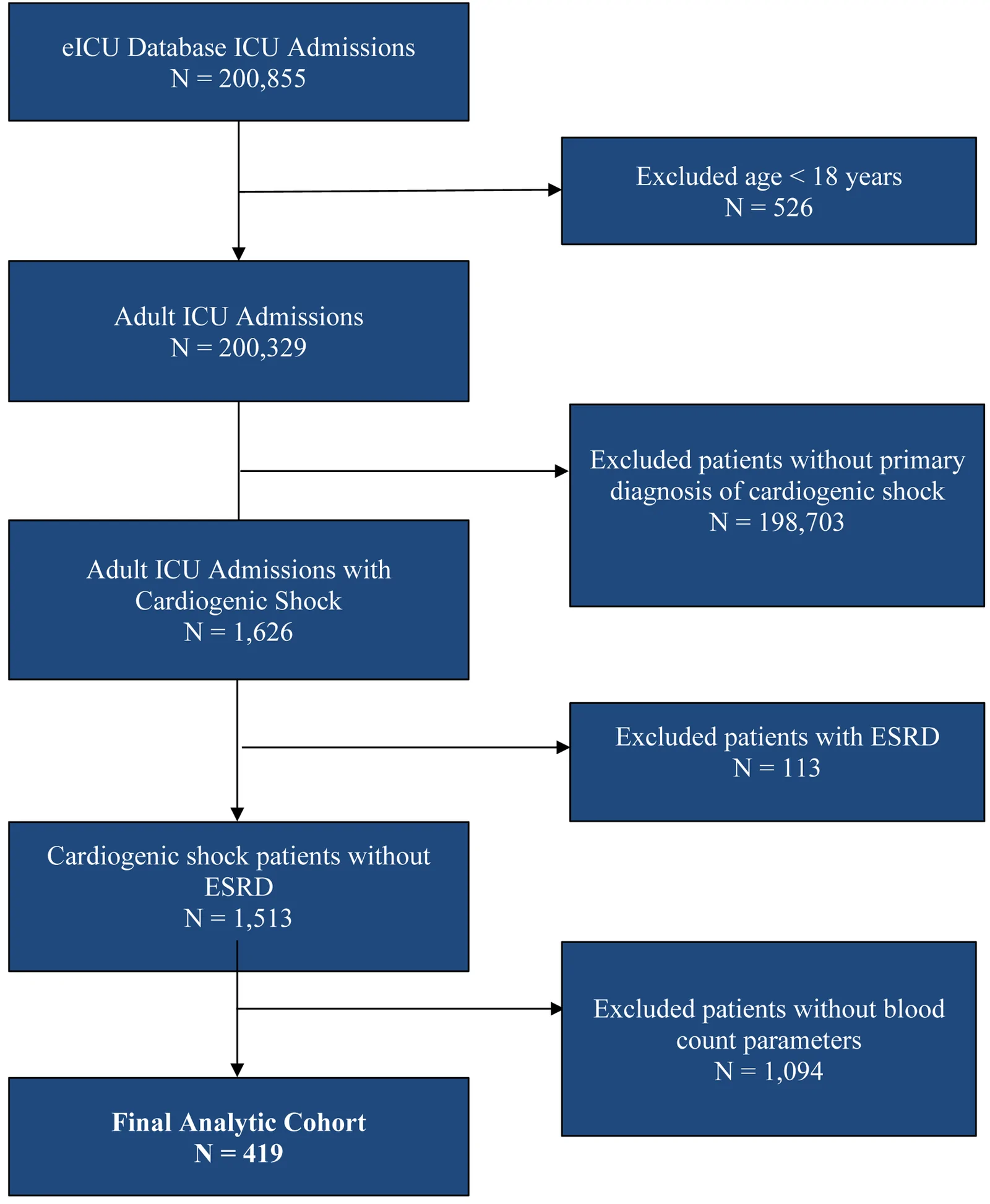
Flowchart of selection of participants. ESRD: end-stage renal disease.

We conducted a retrospective, multicenter cohort study using the eICU Collaborative Research Database, which contains de-identified data from more than 200 intensive care units (ICUs) across the United States, collected between 2014 and 2015 [10]. Adult patients admitted to an ICU with cardiogenic shock were identified using International Classification of Diseases, Ninth Revision, Clinical Modification (ICD-9-CM) codes. Patients missing laboratory components required to calculate the prespecified inflammatory indices were excluded before construction of the analytic cohort because these values were necessary to derive the primary biomarkers evaluated in the study. After the analytic cohort was defined, remaining missingness in secondary covariates was handled using multiple imputation by chained equations (MICE). AKI status was treated as observed and was included in imputation models, but was not itself imputed. For that reason, inflammatory indices were complete in the final analytic cohort by design, whereas other covariates with missing values were retained and imputed. Other inflammatory biomarkers, such as erythrocyte sedimentation rate (ESR), C-reactive protein (CRP), and ferritin, were only recorded sporadically in the database and were excluded from the analysis. Details of cohort assembly and exclusions are shown in Figure 1.

Patients were eligible if they were at least 18 years of age and had an ICU diagnosis of cardiogenic shock. Patients with end-stage renal disease receiving maintenance dialysis before admission were excluded, because AKI cannot be reliably ascertained in this group. Patients were also excluded when the laboratory components required to derive the prespecified inflammatory indices were unavailable, and when no serum creatinine values were recorded during the ICU stay, which would have precluded outcome ascertainment. No exclusions were applied on the basis of illness severity, do-not-resuscitate status, transition to comfort-focused care, or early death, and patients with other potentially fatal conditions such as malignancy, out-of-hospital cardiac arrest, concomitant sepsis, or a requirement for mechanical circulatory support were retained. The cohort therefore includes patients who died early during the ICU course, and the competing risk of death before AKI could be ascertained is acknowledged as a limitation.

Because missing laboratory values led to the exclusion of many patients from the original cardiogenic shock cohort, we evaluated the possibility of selection bias. To address this concern, we compared clinical variables, measures of clinical severity, demographics, need for mechanical ventilation, and renal replacement therapy between the 419 included and the 1,094 excluded subjects. Values are expressed as median with interquartile range (IQR) for continuous variables and n (%) for categorical variables. Outcomes were compared using standardized mean differences (SMD), where SMDs less than 0.10 were considered small, 0.10–0.20 moderate, and greater than 0.20 as meaningful. The differences of SMD between groups of interest are displayed in the Supplementary Material 1 (jocmr.elmerjournals.com).

Demographic variables, comorbidities, intervention, and initial laboratory parameters obtained on admission were extracted from electronic health records during the ICU hospitalization. Inflammatory markers, NLR, PLR, MLR, NPAR, SII, SIRI, and AISI were calculated using standard formulas as follows: NLR = Neutrophil count/Lymphocyte count; PLR = Platelet count/Lymphocyte count; MLR = Monocyte count/Lymphocyte count; SII = (Platelet count × Neutrophil count)/Lymphocyte count; NPAR = (Neutrophil percentage of total white blood cell count (%) × 100)/Albumin (g/dL); SIRI = (Neutrophil count × Monocyte count)/Lymphocyte count; AISI = (Neutrophil count × Platelet count × Monocyte count)/Lymphocyte count.

All inflammatory indices were calculated from the initial complete blood count, differential count, and platelet count obtained at the time of ICU admission, and, for NPAR, from the corresponding admission serum albumin value. These indices therefore represent a single early measurement drawn during the initial ICU laboratory evaluation rather than a serial inflammatory trajectory. Because the eICU Collaborative Research Database aggregates records from more than 200 participating units, a uniform post-admission sampling hour relative to the onset of shock or to pre-ICU resuscitation could not be specified across centers.

AKI is defined based on the 2012 Kidney Disease: Improving Global Outcomes (KDIGO) criteria, as a rise in serum creatinine of ≥ 0.3 mg/dL within 48 h, a rise in serum creatinine of ≥ 1.5 mg/dL above baseline within 7 days, or the need for renal replacement therapy [11]. The baseline serum creatinine was taken as the lowest recorded serum creatinine value within the first 7 days of admission. AKI was the primary outcome and was coded as a binary variable using established creatinine-based criteria and renal replacement therapy status. Because baseline creatinine was derived from laboratory measurements obtained during the same hospitalization period used for AKI ascertainment, this analysis should be interpreted as evaluating factors associated with AKI during ICU hospitalization and early risk stratification, not as a strictly temporally separated incident AKI prediction model. Urine output criteria were not used because reliable urine output data were not consistently available within the database.

As an initial descriptive step, we compared patients with and without AKI. Univariate analyses were performed for baseline characteristics, comorbid conditions, shock-related variables, and laboratory measurements, including all inflammatory indices (AISI, MLR, PLR, NLR, NPAR, SIRI, and SII). Continuous variables were reported as means with standard deviations (SDs) or medians with interquartile ranges. Following that, they were then compared using *t*-tests or appropriate non-parametric methods. Categorical variables were recorded as counts with percentages and compared using χ^2^ or Fisher’s exact tests, as applicable.

Variables considered for multivariable analyses were selected in advance based on clinical likelihood and existing literature. These included demographic characteristics, comorbidities (e.g., chronic kidney disease (CKD), diabetes, heart failure), prognostic modules (e.g., sequential organ failure assessment (SOFA) score), baseline renal function (creatinine and blood urea nitrogen (BUN)), additional routine laboratory parameters, and inflammatory indices. Missing data were assessed for all variables. Several laboratory variables had substantial missing data, while AKI status and most comorbidities were complete. Because the complete-case analysis would have resulted in the removal of a large proportion of the cohort, we used MICE to impute missing covariate values. AKI was treated as observed (not imputed) but was included in the imputation models. Imputation models were specified by variable type, using predictive mean matching for continuous variables and logistic or polytomous regression for categorical variables. Multiple complete datasets were then generated.

We also performed exploratory feature-selection analyses using Boruta and elastic-net regression. Boruta assessed variable importance for AKI classification by comparing observed variables with randomized shadow variables. Elastic-net logistic regression was performed as an exploratory regularization-based analysis in the setting of multiply imputed data. For each imputed dataset, cross-validation identified the optimal penalty parameter. Variable-selection stability was summarized as the proportion of imputed datasets in which each variable was retained. This was very important because several inflammatory indices are derived from overlapping blood-count components and may therefore be collinear. Elastic-net regularization can reduce instability from collinearity by shrinking correlated variables and identifying variables with more stable signals across models. These analyses were exploratory and supportive. However, they were not used as the sole determinant of the final inferential model. The primary multivariable logistic regression model was specified using clinically relevant covariates selected *a priori* according to renal pathophysiology, illness severity, clinical plausibility, and prior literature.

To address the main biological question concerning inflammatory markers, each inflammatory index was evaluated in models adjusted for a base set of clinical covariates (age, sex, CKD, and SOFA score) and subsequently in models additionally adjusted for renal function (creatinine and BUN). Markers were analyzed both on their original scales and in sensitivity analyses, standardized as z-scores such that odds ratios (ORs) reflected the change in odds of AKI per 1-SD increase. Because several inflammatory indices were analyzed, false discovery rate (FDR) correction was applied using the Benjamini-Hochberg procedure. Logistic regression models were used to estimate beta coefficients, standard errors, ORs, 95% confidence intervals (CIs), and P-values. FDR-adjusted q-values were used to control the expected proportion of false positive findings. Statistical significance was interpreted based on these FDR-adjusted q-values.

As creatinine and BUN may not be available at time of risk assessment, we performed an additional sensitivity model excluding creatinine and BUN and included inflammatory markers. Discrimination was assessed using apparent area under the receiver operating characteristic (ROC) curve (AUC) and out-of-fold AUC from elastic-net logistic regression. In addition, for interpretability, a mice imputed regression model employing standardized continuous variables was performed.

Model performance was evaluated using discrimination, calibration, and decision-curve analysis (DCA). Discrimination was measured using the AUC, with estimates summarized across the 20 imputed datasets. Incremental discrimination was assessed by comparing AUC values across the base clinical model, the base model plus renal laboratory variables, and models that also included the inflammatory indices. Calibration was assessed graphically by pooled estimated probabilities across the 20 MICE-imputed datasets by comparing estimated AKI risk with the observed proportion of AKI. DCA was performed to evaluate the potential clinical utility across a range of threshold probabilities by comparing net benefit with treat-all and treat-none strategies.

### Ethical considerations

This study was exempt from institutional review board oversight due to its retrospective design, use of de-identified data, lack of direct patient interaction, and the database security schema, for which re-identification risk was certified as complying with safe harbor standards by an independent privacy expert, Privacert, Cambridge, MA, USA, Health Insurance Portability and Accountability Act Certification No. 1031219-2.

## Results

Among the 419 patients, 65.1% (273) were determined to have AKI using the creatinine-based consensus criteria, while 34.9% (146) did not. Several baseline characteristics were compared between the two groups (Table 1). Patients with AKI had significantly lower serum bicarbonate (18 vs. 20 mmol/L, P = 0.02) and higher initial serum lactate levels (3.6 vs. 2.3 mmol/L, P = 0.02). The baseline serum creatinine was also higher in the AKI group (1.8 vs. 1.2 mg/dL, P < 0.001). Significant differences were also noted in liver enzymes, with higher initial alanine aminotransferase (ALT, 139 vs. 66 U/L, P < 0.001) and aspartate aminotransferase (AST, 231 vs. 88 U/L, P = 0.003) in the AKI group. Initial international normalized ratio (INR) was also higher in patients with AKI (1.7 vs. 1.2, P = 0.003). Notably, the SOFA score (10 vs. 9, P = 0.04) and the lactate to albumin ratio (1.00 vs. 0.78, P = 0.03) were higher in patients with AKI.

**Table 1:** Baseline Clinical Characteristics, Laboratory Parameters, and Univariate Comparisons Between Patients With and Without Acute Kidney Injury in Cardiogenic Shock

Variable	Without AKI (N = 146)	With AKI (N = 273)	P
Age (years)	69 (57, 80)	67 (58, 76)	0.32
BMI (kg/m^2^)	26.8 (23.5, 30.8)	28.4 (24.3, 33.4)	0.06
Initial ALT (U/L)	66 (30, 115)	139 (45, 622)	< 0.001
Bicarbonate (mmol/L)	20 (17, 23)	18 (14, 22)	0.02
Initial AST (U/L)	88 (45, 331)	231 (57, 913)	0.003
Initial lactate (mmol/L)	2.3 (1.5, 4.9)	3.6 (1.9, 7.5)	0.02
Initial INR	1.4 (1.2, 2.1)	1.7 (1.3, 2.9)	0.003
Total bilirubin (mg/dL)	0.8 (0.6, 1.4)	1.0 (0.6, 1.8)	0.08
Creatinine (mg/dL)	1.2 (0.9, 1.8)	1.8 (1.3, 2.9)	< 0.001
Potassium (mmol/L)	4.3 (3.9, 4.9)	4.3 (3.8, 4.9)	0.79
Sodium (mmol/L)	137 (134, 140)	137 (133, 139)	0.16
Platelet count (× 10^3^ cells/µL)	220 (161, 281)	203 (156, 260)	0.16
Monocytes (× 10^3^ cells/µL)	7.0 (5.0, 8.9)	7.0 (5.0, 10.0)	0.87
Hemoglobin (g/dL)	12.1 (10.5, 14.2)	12.3 (10.3, 14.0)	0.68
Albumin(g/dL)	3.4 (3.0, 3.8)	3.3 (2.9, 3.7)	0.16
Lymphocyte count (× 10^3^ cells/µL)	1.5 (0.9, 2.9)	1.4 (0.8, 2.5)	0.25
SII	940 (466, 2,279)	1,200 (491, 2,447)	0.37
AISI (× 10^9^/L)	6,520 (2,970, 14,171)	8,631 (3,272, 15,327)	0.38
NLR	4.6 (2.2, 10.5)	6.1 (3.0, 11.6)	0.13
PLR	135 (76, 233)	143 (79, 241)	0.66
MLR	4.7 (2.5, 7.9)	5.4 (2.4, 8.8)	0.44
SIRI	34.2 (14.8, 66.6)	43.5 (20.9, 76.5)	0.22
Chloride (mmol/L)	102 (96, 105)	101 (96, 104)	0.75
Lactate/albumin ratio	0.78 (0.44, 1.53)	1.0 (0.6, 1.8)	0.03
SOFA on admission	9 (6, 12)	10 (7, 13)	0.04

	Without AKI	With AKI	Odds ratio	95% CI for odds ratio		P
Diabetes	5 (3.4%)	8 (2.9%)	0.86	0.27	2.66	0.79
Cardiomyopathy	2 (1.4%)	9 (3.3%)	2.46	0.53	11.56	0.24
CVA	2 (1.4%)	5 (1.8%)	1.35	0.26	7.04	0.72
CKD	22 (15.1%)	69 (25.4%)	1.92	1.13	3.25	0.01
COPD	15 (10.3%)	23 (8.5%)	0.81	0.41	1.60	0.54
Heart failure	52 (35.6%)	99 (36.4%)	1.03	0.68	1.57	0.87
Hypertension	16 (11.0%)	29 (10.7%)	0.97	0.51	1.85	0.93
Hyperlipidemia	12 (8.2%)	10 (3.7%)	0.43	0.18	1.01	0.04
Malignancy	5 (3.4%)	8 (2.9%)	0.86	0.27	2.66	0.79
ARB	2 (1.4%)	11 (4.0%)	3.03	0.66	13.88	0.13
ACEi	20 (13.7%)	27 (9.9%)	0.69	0.38	1.29	0.24
Diuretics	33 (22.6%)	88 (32.4%)	1.64	1.03	2.60	0.04
IABP	17 (11.6%)	42 (15.4%)	1.38	0.76	2.52	0.294
Vasopressors	110 (75.3%)	230 (84.2%)	1.75	1.06	2.88	0.03
Primary CABG	1 (0.7%)	1 (0.4%)	0.52	0.03	8.59	0.652
Emergent CABG	1 (0.7%)	2 (0.7%)	1.07	0.10	11.90	0.96
Ethnicity						
Caucasian	113 (77.4%)	208 (76.2%)				0.27
African Americans	15 (10.3%)	28 (10.3%)				
Asians	3 (2.1%)	8 (2.9%)				
Hispanics	11 (7.5%)	11 (4.0%)				
Other	4 (2.7%)	18 (16.6%)				
Acute MI	40 (27.4%)	91 (33.3%)	1.33	0.85	2.06	0.21
PCI	14 (9.6%)	25 (9.2%)	0.95	0.48	1.89	0.89
Male sex	85 (58.2%)	167 (61.2%)	1.12	0.75	1.70	0.56
Mechanical ventilation	92 (63.0%)	190 (69.9%)	1.36	0.89	2.08	0.16

ACEi: angiotensin-converting enzyme inhibitor; AISI: aggregate index of systemic inflammation; AKI: acute kidney injury; ALT: alanine transaminase; ARB: angiotensin receptor blocker; AST: aspartate transaminase; BMI: body mass index; BUN: blood urea nitrogen; CABG: coronary artery bypass graft; CI: confidence interval; CKD: chronic kidney disease; COPD: chronic obstructive pulmonary disease; CVA: cerebrovascular accident; HD: hemodialysis; IABP: intra-aortic balloon pump; INR: international normalized ratio; MI: myocardial infarction; MLR: monocyte to lymphocyte ratio; NLR: neutrophil to lymphocyte ratio; NPAR: neutrophil percentage to albumin ratio; PCI: percutaneous coronary intervention; PLR: platelet to lymphocyte ratio; RRT: renal replacement therapy; SII: systemic immune-inflammation index; SIRI: systemic inflammation response index; SOFA: sequential organ failure assessment.

The inflammatory indices, including the SII (1,200 vs. 940, P = 0.37), AISI (8,631 vs. 6,520, P = 0.38), NLR (6.1 vs. 4.6, P = 0.13), PLR (143 vs. 135, P = 0.66), MLR (5.4 in AKI vs. 4.7, P = 0.44), and SIRI (43.5 vs. 34.2, P = 0.22) were higher in the AKI groups but without achieving statistical significance.

The analysis assessing the effects of comorbid conditions on the incidence indicated that a diagnosis of CKD was associated with higher odds of developing AKI (OR = 1.92, 95% CI: 1.13–3.25, P = 0.01). In contrast, our data suggest that a diagnosis of hyperlipidemia was associated with lower odds of developing AKI (OR = 0.43, 95% CI: 0.18–1.01, P = 0.04). Other variables associated with higher odds of developing AKI included the use of diuretics (OR = 1.64, 95% CI: 1.03–2.60, P = 0.04) and vasopressors (OR = 1.75, 95% CI: 1.06–2.88, P = 0.03).

Substantial missingness was observed for certain laboratory values as mentioned in Supplementary Material 1 (jocmr.elmerjournals.com). Renal markers, including creatinine and BUN, had 6.7% missing data. Data for AKI status, demographics (age and sex), and all inflammatory indices (SIRI, SII, NLR, PLR, MLR, AISI, and NPAR) were 100% complete. As many variables had more than 5% missing data, MICE was used to impute missing values across 20 imputed datasets.

The observed imbalances in clinical variables, measures of clinical severity, demographics, need for mechanical ventilation, and renal replacement therapy between the 419 included subjects and the 1,094 excluded subjects were similar between the groups, with SMDs ranging from small to modest (Supplementary Material 1, jocmr.elmerjournals.com). These results support broad comparability between groups. Although the findings support the conclusion that the two groups are broadly similar, residual selection bias due to differences in monitoring intensity, laboratory evaluation patterns, and other unmeasured clinical variables cannot be excluded.

Using the Boruta algorithm (Supplementary Materials 2 and 3, jocmr.elmerjournals.com), we identified variables with greatest importance for AKI classification. AISI, creatinine, ALT, AST, BUN, INR, total bilirubin, lactate/albumin, and SOFA score were classified as confirmed. Inflammatory indices like SII, monocytes, and SIRI showed evidence of importance, whereas NLR, MLR, PLR, and NPAR were classified as rejected.

In exploratory elastic-net analysis performed in the setting of multiply imputed data, renal function variables demonstrated the greatest selection stability (Supplementary Material 4, jocmr.elmerjournals.com). BUN and creatinine were retained in all imputed datasets, and ALT was retained in 95% of imputed datasets. Variable-selection frequency across imputed datasets is shown in Supplementary Material 5 (jocmr.elmerjournals.com). AST and lactate/albumin ratio showed low selection frequency and were not retained as stable variables. Inflammatory indices were not consistently retained in elastic-net models, supporting the finding that they did not provide stable incremental signal beyond established renal and clinical variables.

In the primary pooled multivariable model, baseline creatinine was the only independent laboratory variable significantly associated with AKI (OR 1.63, 95% CI 1.16–2.3, P = 0.005). In this adjusted model, other clinical factors, including age, sex, and baseline CKD, did not reach statistical significance (Table 2).

**Table 2:** Primary Multivariable Logistic Regression Evaluating Variables Associated With Acute Kidney Injury

Variables	OR	CI		P value
Intercept	0.72	0.22	2.35	0.59
Creatinine	1.63	1.16	2.3	0.01
BUN	1.01	0.99	1.02	0.21
ALT	1.00	1.00	1.00	0.18
SOFA score	1.00	0.95	1.06	0.93
Age	0.99	0.98	1.01	0.43
Sex (male)	0.97	0.63	1.51	0.90
CKD	0.94	0.5	1.77	0.85

Covariates in this model were specified a priori and comprised baseline serum creatinine, blood urea nitrogen, alanine transaminase, SOFA score on admission, age, sex, and a history of chronic kidney disease. Creatinine and blood urea nitrogen were included as direct measures of baseline renal function, alanine transaminase as a marker of hepatic congestion or ischemic hepatic injury reflecting hemodynamic compromise, SOFA score as a measure of global illness severity, age and sex as demographic confounders, and chronic kidney disease as an indicator of preexisting renal vulnerability. Estimates are pooled across 20 MICE-imputed datasets. OR for ALT are rounded off to 1.00 as the estimated effect size was extremely small per 1-unit increase. ALT: alanine transaminase; BUN: blood urea nitrogen; CI: confidence interval; CKD: chronic kidney disease; OR: odds ratio; SOFA: sequential organ failure assessment.

However, the sensitivity analysis (Table 3), which excluded baseline creatinine and BUN, showed that CKD was significantly associated with AKI (OR 1.94, 95% CI 1.14–3.32, P = 0.016).

**Table 3:** Sensitivity Analysis: Multivariable Logistic Regression Model for Acute Kidney Injury Excluding Baseline Renal Function Variables

Variables	OR	CI		P value
Intercept	1.23	0.39	3.85	0.72
ALT	1.00	1.00	1.00	0.14
SOFA score	1.03	0.98	1.09	0.27
Age	1.00	0.99	1.01	0.68
Sex (male)	1.11	0.73	1.70	0.62
CKD	1.95	1.14	3.34	0.02

This model was adjusted for the same a priori covariates as the primary model with the exception of baseline serum creatinine and blood urea nitrogen, which were deliberately omitted; adjustment therefore comprised alanine transaminase, SOFA score on admission, age, sex, and chronic kidney disease. Renal laboratory values were removed because they are already abnormal, at the time of early risk assessment, which allows the independent contribution of preexisting chronic kidney disease to be estimated. Estimates are pooled across 20 MICE-imputed datasets. ALT: alanine transaminase; CI: confidence interval; CKD: chronic kidney disease; OR: odds ratio; SOFA: sequential organ failure assessment.

After applying FDR correction, none of the inflammatory indices demonstrated a statistically significant association with AKI. In model A (adjusted for clinical covariates), the FDR-adjusted q-values ranged from 0.960 to 0.974, while in model B (adjusted for clinical covariates and renal function), all indices had q-values of 0.776. The effect estimates for all inflammatory markers were very small, and the corresponding CIs crossed unity, indicating that they were not independently associated with AKI after correction for multiple comparisons (Table 4).

**Table 4:** Association Between Inflammatory Indices and Acute Kidney Injury Using Multivariable Logistic Regression Models With False Discovery Rate (FDR) Adjustment

Index	OR	CI		P value
Model A: adjusted for clinical covariate				
AISI	1.00	0.82	1.23	0.98
MLR	1.15	0.91	1.45	0.26
NLR	1.01	0.82	1.25	0.89
NPAR	1.06	0.86	1.32	0.56
PLR	1.09	0.87	1.38	0.45
SII	0.98	0.80	1.21	0.88
SIRI	1.06	0.85	1.31	0.6
Lactate/albumin ratio	1.21	0.82	1.79	0.32
Model B: adjusted for clinical and renal function				
AISI	0.96	0.77	1.19	0.68
MLR	1.04	0.82	1.32	0.72
NLR	0.95	0.76	1.18	0.64
NPAR	0.93	0.74	1.18	0.56
PLR	1.07	0.85	1.34	0.56
SII	0.97	0.79	1.20	0.78
SIRI	0.95	0.75	1.19	0.63
Lactate/albumin ratio	1.11	0.75	1.63	0.6

Model A was adjusted for the a priori clinical covariates age, sex, chronic kidney disease, and SOFA score on admission. Model B was additionally adjusted for baseline serum creatinine and blood urea nitrogen, in order to test whether any association was independent of conventional measures of renal function. Each index was entered separately, and odds ratios are expressed per 1-SD increase in the standardized index. q-values were derived using the Benjamini-Hochberg false discovery rate procedure across the indices tested. AISI: aggregate index of systemic inflammation; CI: confidence interval; MLR: monocyte to lymphocyte ratio; NLR: neutrophil to lymphocyte ratio; OR: odds ratio; PLR: platelet to lymphocyte ratio; NPAR: neutrophil percentage to albumin ratio; SII: systemic immune-inflammation index; SIRI: systemic inflammation response index.

**Figure 2:**
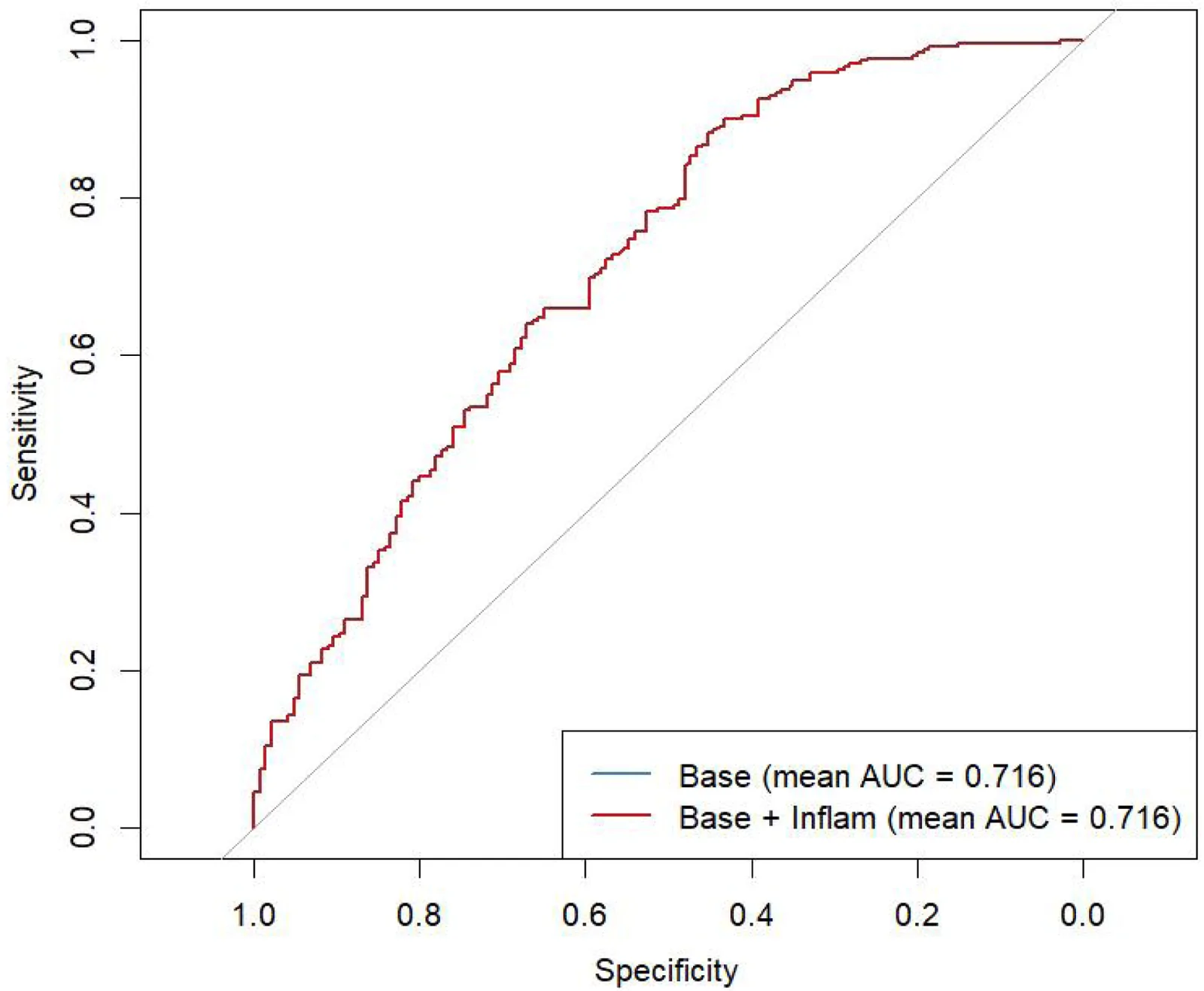
Receiver operating characteristic curve comparing base model with the combined inflammatory-index model.

The base clinical model demonstrated modest discrimination for AKI, with a mean AUC of 0.594. Inclusion of baseline renal laboratory markers significantly improved model performance, increasing the AUC to 0.704. Although several inflammatory indices, including MLR, PLR, and SIRI, produced small increases in AUC when added to the clinical model alone, they did not provide meaningful incremental discrimination once renal function variables were included (Table 5). The final pooled model, which included both clinical variables and all inflammatory indices, had a stable AUC of 0.72 (95% CI 0.70–0.73). Table 5 evaluates the incremental discrimination of each inflammatory index added individually to the base clinical model and to the base clinical plus renal model. In contrast, Figure 2 shows the pooled ROC analysis for the combined inflammatory index model, in which all inflammatory indices were added together.

**Table 5:** Incremental Discrimination of Individual Inflammatory Indices for AKI During ICU Hospitalization (AUC Analysis)

Inflammatory index	Base AUC (mean SD)	Base + index AUC (mean SD)	ΔAUC (mean SD)	Base + renal AUC (mean SD)	Base + renal + index AUC (mean SD)	ΔAUC (mean SD)
SIRI	0.594 (0.001)	0.598 (0.002)	0.0040 (0.0006)	0.704 (0.009)	0.704 (0.009)	0.0002 (0.0006)
MLR	0.594 (0.001)	0.603 (0.002)	0.0088 (0.0005)	0.704 (0.009)	0.703 (0.009)	−0.0004 (0.0004)
NLR	0.594 (0.001)	0.595 (0.002)	0.0008 (0.0003)	0.704 (0.009)	0.703 (0.009)	−0.0007 (0.0006)
PLR	0.594 (0.001)	0.598 (0.002)	0.0040 (0.0008)	0.704 (0.009)	0.703 (0.009)	−0.0008 (0.0006)
AISI	0.594 (0.001)	0.594 (0.002)	0.0001 (0.0003)	0.704 (0.009)	0.703 (0.009)	−0.0009 (0.0006)
NPAR	0.594 (0.001)	0.598 (0.002)	0.0039 (0.0006)	0.704 (0.009)	0.703 (0.009)	−0.0009 (0.0007)
SII	0.594 (0.001)	0.594 (0.002)	−0.0002 (0.0002)	0.704 (0.009)	0.702 (0.009)	−0.0011 (0.0006)

The base model was adjusted for the a priori clinical covariates age, sex, chronic kidney disease, and SOFA score on admission, chosen because they are routinely available at the time of early risk assessment and represent demographic risk, preexisting renal vulnerability, and global illness severity. The base plus renal model additionally included baseline serum creatinine and blood urea nitrogen, so that incremental discrimination could be judged against conventional renal function testing. Each inflammatory index was added individually to both models, because several indices share common blood-count components and entering them simultaneously would introduce collinearity. All AUC values are means with standard deviations across 20 MICE-imputed datasets. AISI: aggregate index of systemic inflammation; AKI: acute kidney injury; AUC: area under the curve; ICU: intensive care unit; MLR: monocyte to lymphocyte ratio; NLR: neutrophil to lymphocyte ratio; OR: odds ratio; PLR: platelet to lymphocyte ratio; NPAR: neutrophil percentage to albumin ratio; SD: standard deviation; SII: systemic immune-inflammation index; SIRI: systemic inflammation response index.

In the sensitivity analysis which excluded creatinine and BUN, the addition of inflammatory markers demonstrated no improvement in discrimination. The apparent AUC was 0.62 for the non-renal clinical model and 0.63 after the addition of inflammatory markers. The corresponding out-of-fold AUCs were 0.52 in both models. In the accompanying MICE-pooled logistic regression, adjusted for non-renal clinical covariates, no inflammatory markers were statistically associated with AKI. Full regression results are provided in Supplementary Materials 6 and 7 (jocmr.elmerjournals.com).

**Figure 3:**
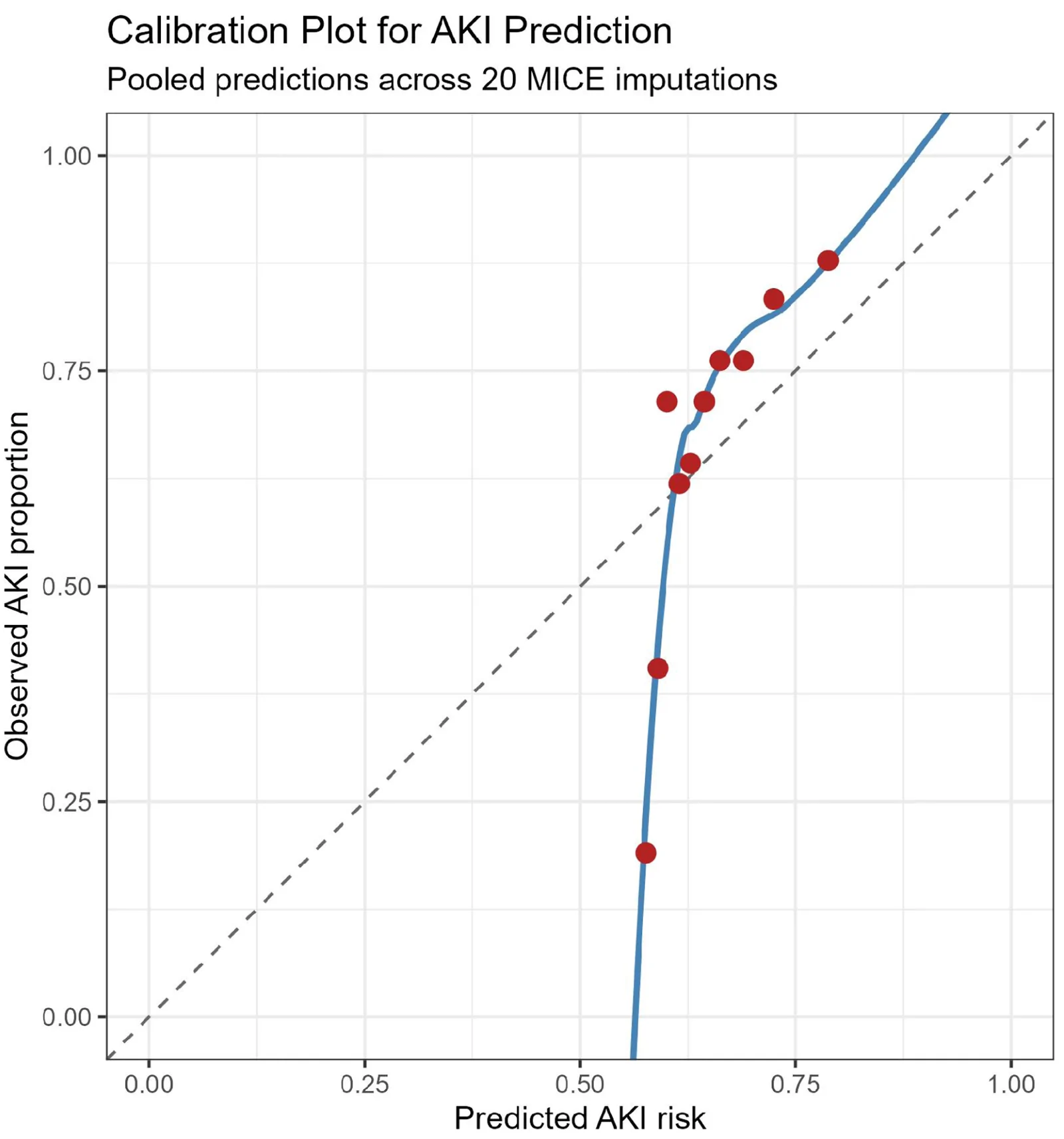
Calibration plot for AKI risk estimation, comparing estimated AKI risk with observed AKI proportion using pooled estimated probabilities across 20 MICE-imputed datasets. The dashed diagonal line indicates perfect calibration. AKI: acute kidney injury.

**Figure 4:**
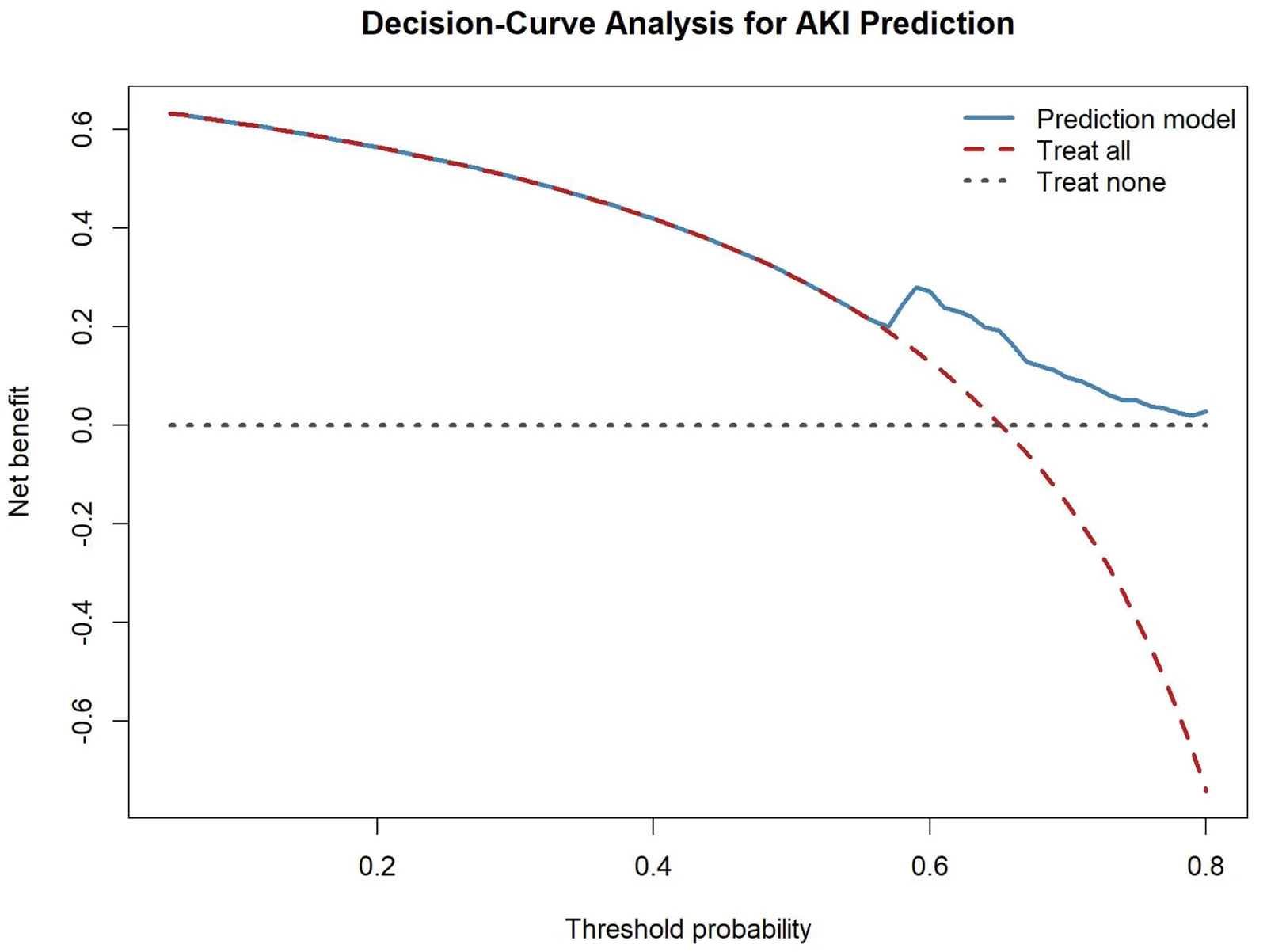
Decision-curve analysis for AKI risk estimation, comparing the net benefit of the AKI risk model with treat-all and treat-none strategies across threshold probabilities. AKI: acute kidney injury.

Additionally, calibration and DCA were performed to further evaluate model performance. The calibration plot based on pooled estimated probabilities across 20 MICE-imputed datasets demonstrated the relationship between estimated AKI risk and observed AKI proportion (Fig. 3). Estimated probabilities were concentrated in a relatively narrow range, reflecting the high AKI prevalence in the cohort and the modest discriminatory ability of the model. The DCA suggested that the model may provide net benefit compared with treat-none and treat-all strategies across selected threshold probabilities (Fig. 4).

## Discussion

In this multicenter ICU cohort of patients with cardiogenic shock, AKI occurred in approximately two-thirds of the patients, underscoring how common renal complications are in this high-risk cardiogenic shock population. The occurrence of AKI was associated with markers of systemic illness severity and baseline renal dysfunction on univariate analysis. However, inflammatory indices derived from routine hematologic parameters were not independently associated with AKI and added very little to risk discrimination once standard clinical and biochemical variables were already considered. Importantly, these markers also did not remain significant after adjustment for multiple comparisons using FDR correction, and none were retained in the elastic-net feature selection models, which makes it unlikely that these indices add a stable signal in this cohort. Remaining covariate missingness was handled with MICE.

Baseline creatinine was the strongest variable associated with AKI in the primary multivariable model. This aligns with prior studies showing that patients with reduced baseline renal function are particularly susceptible to renal injury during episodes of circulatory failure [12]. In the setting of cardiogenic shock, reduced cardiac output leads to renal hypoperfusion [13], while venous congestion further increases renal interstitial pressure and impairs glomerular filtration [14]. Together, these mechanisms predispose patients with underlying renal dysfunction to rapid deterioration in kidney function [15].

This point needs careful interpretation because baseline serum creatinine was defined as the lowest recorded creatinine value within the first 7 days of hospitalization, while AKI was defined using creatinine-based KDIGO criteria during the ICU stay. Accordingly, some degree of temporal overlap between variable measurement and outcome ascertainment is unavoidable, and part of the observed association between baseline renal function and AKI may reflect renal dysfunction already evolving at presentation. For that reason, our results are best viewed as an assessment of association and incremental discrimination during the ICU stay, not as a strict incident-AKI prediction model with a clean temporal separation from baseline assessment.

The role of underlying renal vulnerability was further supported in our sensitivity analysis, excluding creatinine and BUN. In this model, CKD was independently associated with AKI. These findings indicate that preexisting renal impairment predisposes patients to kidney injury even in the absence of contemporaneous laboratory markers of renal dysfunction and support the concept that AKI in cardiogenic shock frequently represents the interplay between acute hemodynamic insult and chronic renal vulnerability [16].

Patients with AKI displayed several laboratory abnormalities consistent with more severe physiologic disturbance. Higher serum lactate levels, lower bicarbonate levels, and increased lactate/albumin ratios were observed among AKI patients, which suggests more profound systemic hypoperfusion and metabolic acidosis. In addition, elevated transaminases and INR likely reflect hepatic congestion or ischemic injury secondary to reduced cardiac output and venous congestion.

The higher SOFA scores in the AKI group further support the notion that kidney injury in cardiogenic shock develops in the setting of broader multiorgan dysfunction [17]. Overall, these findings suggest that AKI in cardiogenic shock is largely a consequence of global hemodynamic compromise and organ hypoperfusion rather than an isolated renal pathology or a primary inflammatory process [18].

We specifically wanted to test whether inflammatory indices derived from routine hematologic parameters were independently associated with AKI and provided incremental discrimination beyond established clinical and renal variables in patients with cardiogenic shock. Although several indices, including NLR, PLR, MLR, SIRI, and SII, were numerically higher among patients who developed AKI, none showed statistically significant associations in multivariable analyses. Additionally, all associations remained non-significant after adjustment for multiple comparisons using FDR correction.

These findings suggest that inflammatory indices may have limited incremental value for AKI risk stratification in this population [18]. Although systemic inflammation plays a role in the pathophysiology of many critical illnesses, AKI in cardiogenic shock appears to be largely mediated by hemodynamic and perfusion-related mechanisms, including reduced renal blood flow, venous congestion, and neurohumoral activation [19]. Peripheral blood-count-based inflammatory markers may simply be too nonspecific to capture the more complex heart–kidney physiology driving AKI in cardiogenic shock [20].

Several explanations could be considered before concluding that inflammation is unimportant in this setting. First, the indices were derived from a single blood draw obtained at the beginning of the ICU stay, and many patients had already received revascularization, vasoactive support, or mechanical circulatory support before that sample was taken, so early stabilization may have attenuated the measurable inflammatory signal. Second, the median SOFA score of 9 to 10 and the observed lactate distribution suggest a cohort with moderate rather than uniformly profound shock, and the inflammatory contribution to organ injury appears most pronounced in the most severe shock phenotypes [21, 22]. Third, the pattern of elevated transaminases, prolonged INR, and higher lactate/albumin ratios in the AKI group is more consistent with congestion- and hypoperfusion-driven renal injury than with a primary immune-mediated process, and congestion is not captured by blood-count ratios. Direct assessment of renal venous congestion using Doppler or the venous excess ultrasound approach has been associated with AKI in cardiac populations and was not available in this database [23, 24]. Fourth, blood-count-derived ratios are indirect surrogates of the inflammatory state and are influenced by catecholamine-induced leukocyte redistribution, corticosteroid exposure, transfusion, and hemodilution, so they may lack the sensitivity of direct cytokine measurement; interleukin-6 and related cytokines have been associated with outcome in cardiogenic shock and with AKI in cardiac surgical cohorts [25–27]. Finally, inflammation in cardiogenic shock evolves over hours to days, so a single early measurement may miss the window in which inflammatory activation becomes relevant to renal injury; biomarker-driven subphenotyping and tubular-injury markers such as neutrophil gelatinase-associated lipocalin and proenkephalin have shown greater promise than blood-count ratios and represent a more informative direction for future work [21, 28]. Taken together, these considerations indicate that routinely available blood-count indices are inadequate tools for AKI risk stratification in cardiogenic shock, rather than that inflammation is irrelevant to its pathogenesis.

Our discrimination analyses further support this interpretation. The base clinical model demonstrated discrimination, and adding inflammatory indices yielded only marginal improvements in the AUC. Even with the renal laboratory parameters included in the base model, inflammatory markers provided minimal additional incremental discriminatory value. Overall, these results suggest that routine inflammatory indices may not meaningfully improve risk stratification beyond established clinical and laboratory variables [29].

To strengthen model-performance reporting, we added calibration and DCA. The calibration plot showed the relationship between estimated and observed AKI risk across pooled estimated probabilities from the imputed datasets. However, the estimated probabilities were clustered within a limited range, consistent with the modest discrimination of the model and the high prevalence of AKI in this cohort. DCA suggested potential net benefit of the model across selected threshold probabilities, particularly when compared with a treat-none strategy and at higher thresholds compared with a treat-all strategy. These findings should be interpreted cautiously given the retrospective design and lack of external validation.

### Strengths and limitations

One of the several strengths of this study is the use of a large, multicenter ICU database, which enabled the evaluation of a heterogeneous cohort of patients with cardiogenic shock and ultimately enhanced generalizability. Traditional regression models combined with machine learning–based feature selection provided complementary approaches to identifying variables associated with AKI. Multiple imputation was used to address missing data while preserving statistical power.

However, a few limitations warrant consideration. First, the study’s retrospective design limits causal inference and may introduce confounding. Second, because baseline creatinine was derived from laboratory measurements obtained during the same hospitalization period used for AKI ascertainment, some degree of temporal overlap and outcome leakage is possible. Accordingly, these models should not be interpreted as strict incident-AKI prediction models. Third, although baseline inflammatory indices were assessed, their dynamic changes during the ICU course were not evaluated, which may have provided additional prognostic insight. Fourth, institutional variations in clinical management, timing of laboratory testing, and AKI detection may have influenced observed associations. Fifth, all inflammatory indices were derived from a single admission blood draw, and a uniform sampling time relative to the onset of shock and to pre-ICU resuscitation could not be defined across participating centers, so timing-related attenuation of the inflammatory signal cannot be excluded. Sixth, although the analytic cohort of 419 patients with 273 events was adequate for the modest number of *a priori* covariates in the primary model, statistical power to detect small independent associations for the inflammatory indices and to estimate small increments in discrimination precisely was limited. Contemporary sample-size guidance for prediction-model development indicates that considerably larger cohorts are required to estimate small changes in the AUC with confidence [30]. We therefore cannot exclude a genuine but small association between blood-count-derived inflammatory indices and AKI that this cohort was underpowered to detect, and our negative findings are best interpreted as an absence of clinically useful incremental discrimination at this sample size rather than as definitive evidence of no association. Seventh, the range of AKI risk factors examined was necessarily restricted to variables reliably captured in a retrospective critical care database. Hemodynamic data such as cardiac index, filling pressures, and central venous pressure, echocardiographic and Doppler measures of renal and hepatic congestion, cumulative fluid balance, vasopressor and inotrope dose intensity, iodinated contrast exposure, nephrotoxic drug administration, the type and duration of mechanical circulatory support, urine output, and direct cytokine or tubular-injury biomarkers were unavailable. A more comprehensive multidimensional assessment incorporating these hemodynamic, congestion, iatrogenic, and biomarker domains would be required to characterize the mechanisms of AKI in cardiogenic shock, and the present study should therefore be regarded as an evaluation of routinely available blood-count indices rather than a complete account of AKI risk in this population. Although calibration and DCA were added to improve model-performance reporting, the model was not externally validated, and optimism-corrected performance estimates were not fully established. Estimated probabilities were also concentrated within a relatively narrow range, which may limit assessment of calibration across the full spectrum of AKI risk. Finally, selection bias is possible because patients lacking laboratory components required to calculate inflammatory indices were excluded before imputation. Therefore, the findings applied to our complete-index analytic cohort rather than the entire source population of patients with cardiogenic shock.

### Conclusions

AKI occurred in approximately two-thirds of the patients in this critically ill patient cohort with cardiogenic shock. AKI was associated primarily with baseline renal dysfunction and markers of systemic illness severity. Baseline creatinine emerged as the strongest variable associated with AKI, while an increased risk association with CKD was observed when renal laboratory markers were omitted, emphasizing the significance of underlying renal vulnerability in this population.

Despite the prior interest in inflammatory indices as prognostic biomarkers, our findings indicate that hematologic inflammatory markers, including NLR, PLR, MLR, SIRI, SII, AISI, and NPAR, were not independently associated with AKI after multivariable adjustment and FDR correction. They also did not demonstrate meaningful incremental discrimination beyond established renal and clinical variables associated with AKI during ICU hospitalization. With the inclusion of renal function measures, model discrimination was markedly improved, whereas inflammatory indices had minimal effect. Calibration and DCA provided additional context for model performance; however, these findings remain exploratory and require external validation before clinical application. These results should not be interpreted as evidence that inflammation is unimportant in the pathogenesis of AKI in cardiogenic shock. Our analysis was restricted to indices derived from a single early complete blood count and did not include direct measures of inflammatory activation such as C-reactive protein or interleukin-6, nor markers of tubular injury, and the size of the cohort limited power to detect small associations. The findings therefore indicate that routinely available blood-count-derived indices are unlikely to be clinically useful for early AKI risk stratification in cardiogenic shock, whereas the biological contribution of inflammation remains plausible and unresolved [21, 25]. Future studies incorporating longitudinal renal function, urine-output data, hemodynamic parameters, and markers of renal congestion may better clarify AKI risk stratification in cardiogenic shock.

## Supplementary Material

Suppl 1 Comparison of included analytic cohort and excluded cardiogenic shock patients.

Suppl 2 Boruta Stability All Variables.

Suppl 3 Boruta variable-importance distributions for acute kidney injury classification. Boxplots summarize variable-importance scores across Boruta iterations. Green indicates confirmed variables, red indicates rejected variables, and blue indicates shadow features used as reference comparators.

Suppl 4 Exploratory Elastic-net Model Performance Across MICE-Imputed Datasets.

Suppl 5 Exploratory Elastic-Net Variable Selection Frequency Across MICE-Imputed Datasets.

Suppl 6 MICE imputed pooled multivariable logistic regression analysis excluding creatinine and BUN.

Suppl 7 Sensitivity Analysis of Inflammatory Markers for AKI discrimination After Excluding Renal Function Markers.

## Data Availability

The data analyzed in this study are available through the eICU Collaborative Research Database after completion of required training and data-use approval. No identifiable patient-level data were used in this study.
