# Supplementary material for: Blood Count-Based Inflammatory Indices Do Not Improve Prediction of Acute Kidney Injury in Cardiogenic Shock
Source: J Clin Med Res. 2026 Aug 26;18(8):528–41. doi: 10.14740/jocmr6666 (PMC13568768; doi:10.14740/jocmr6666)
Supplement: Supplementary file 1 [file jocmr-18-08-528-s001.docx]

**Suppl 1.** Comparison of included analytic cohort and excluded cardiogenic shock patients

| **Characteristic** | **Included cohort (n=419)** | **Excluded cohort (n=1,094)** | **SMD** |
| --- | --- | --- | --- |
| **Demographics** |  |  |  |
| Age, years | 67.00 [58.00, 77.00] | 67.00 [56.00, 76.00] | 0.059 |
| Male sex | 252 (60.1%) | 708 (64.7%) | 0.095 |
| Body mass index, kg/m² | 27.60 [24.05, 32.48] | 28.20 [24.20, 33.80] | 0.042 |
| Ethnicity: Caucasian | 321 (76.6%) | 838 (76.6%) | 0.000 |
| Ethnicity: African American | 43 (10.3%) | 122 (11.2%) | 0.029 |
| Ethnicity: Asian | 11 (2.6%) | 19 (1.7%) | 0.061 |
| Ethnicity: Hispanic | 22 (5.3%) | 43 (3.9%) | 0.063 |
| Ethnicity: Native American | 2 (0.5%) | 6 (0.5%) | 0.010 |
| Ethnicity: Other/Unknown | 19 (4.5%) | 66 (6.0%) | 0.067 |
| **Severity and laboratory values** |  |  |  |
| SOFA score | 10.00 [7.00, 13.00] | 9.00 [6.00, 12.00] | 0.115 |
| BUN, mg/dL | 35.00 [22.00, 52.50] | 33.00 [21.00, 50.00] | 0.109 |
| Creatinine, mg/dL | 1.63 [1.10, 2.54] | 1.46 [0.97, 2.20] | 0.124 |
| Lactate, mmol/L | 3.20 [1.77, 6.62] | 3.20 [1.60, 7.20] | 0.021 |
| Potassium, mmol/L | 4.30 [3.80, 4.90] | 4.20 [3.70, 4.80] | 0.115 |
| Sodium, mmol/L | 137.00 [134.00, 140.00] | 137.50 [134.00, 140.00] | 0.063 |
| Hematocrit, % | 37.40 [32.00, 42.80] | 37.30 [31.45, 42.20] | 0.081 |
| Hemoglobin, g/dL | 12.20 [10.50, 14.10] | 12.10 [10.20, 14.03] | 0.056 |
| Albumin, g/dL | 3.30 [2.90, 3.80] | 3.20 [2.80, 3.62] | 0.198 |
| **Comorbidities** |  |  |  |
| Hypertension | 45 (10.8%) | 117 (10.7%) | 0.002 |
| Diabetes | 13 (3.1%) | 30 (2.7%) | 0.022 |
| COPD | 38 (9.1%) | 75 (6.9%) | 0.083 |
| Hyperlipidemia | 22 (5.3%) | 67 (6.1%) | 0.037 |
| Cerebrovascular disease | 7 (1.7%) | 38 (3.5%) | 0.114 |
| Malignancy | 13 (3.1%) | 33 (3.0%) | 0.005 |
| Chronic renal disease | 91 (21.8%) | 196 (17.9%) | 0.097 |
| Cardiomyopathy | 11 (2.6%) | 57 (5.2%) | 0.133 |
| Heart failure | 151 (36.1%) | 378 (34.6%) | 0.033 |
| Treatments and organ support |  |  |  |
| Vasoactive drug use | 339 (81%) | 839 (77%) | 0.108 |
| Diuretic use | 121 (28.9%) | 323 (29.5%) | 0.013 |
| ACE inhibitor use | 47 (11.2%) | 101 (9.2%) | 0.066 |
| ARB use | 13 (3.1%) | 22 (2.0%) | 0.070 |
| Mechanical ventilation | 282 (67.5%) | 746 (68.2%) | 0.016 |
| Dialysis/RRT | 55 (13.1%) | 168 (15.4%) | 0.064 |
| Outcomes/disposition |  |  |  |
| Unit mortality | 115 (27.4%) | 281 (25.7%) | 0.040 |
| Hospital mortality | 146 (34.8%) | 345 (31.5%) | 0.070 |
| ICU length of stay, days | 3.4 [1.6, 6.7] | 3.7 [1.77, 7] | 0.113 |
