# Supplementary material for: Blood Count-Based Inflammatory Indices Do Not Improve Prediction of Acute Kidney Injury in Cardiogenic Shock
Source: J Clin Med Res. 2026 Aug 26;18(8):528–41. doi: 10.14740/jocmr6666 (PMC13568768; doi:10.14740/jocmr6666)
Supplement: Supplementary file 2 [file jocmr-18-08-528-s002.docx]

**Suppl 2:** Boruta Stability All Variables

| **Rank** | **Variable** | **Decision** | **Confirmed %** | **Tentative %** | **Rejected %** |
| --- | --- | --- | --- | --- | --- |
| 1 | AISI | Confirmed | 100 | 0 | 0 |
| 2 | Creatinine | Confirmed | 100 | 0 | 0 |
| 3 | ALT | Confirmed | 100 | 0 | 0 |
| 4 | AST | Confirmed | 100 | 0 | 0 |
| 5 | BUN | Confirmed | 100 | 0 | 0 |
| 6 | INR | Confirmed | 100 | 0 | 0 |
| 7 | Total bilirubin | Confirmed | 100 | 0 | 0 |
| 8 | Lactate/Albumin | Confirmed | 100 | 0 | 0 |
| 9 | Sofa score | Confirmed | 100 | 0 | 0 |
| 10 | Sodium | Confirmed | 95 | 0 | 5 |
| 11 | SII | Confirmed | 90 | 0 | 10 |
| 12 | SIRI | Confirmed | 90 | 0 | 10 |
| 13 | Monocytes | Confirmed | 90 | 0 | 10 |
| 14 | NLR | Rejected | 50 | 0 | 50 |
| 15 | Chloride | Rejected | 0 | 0 | 100 |
| 16 | IABP | Rejected | 0 | 0 | 100 |
| 17 | MLR | Rejected | 0 | 0 | 100 |
| 18 | NPAR | Rejected | 0 | 0 | 100 |
| 19 | PLR | Rejected | 0 | 0 | 100 |
| 20 | Admission BMI | Rejected | 0 | 0 | 100 |
| 21 | Age | Rejected | 0 | 0 | 100 |
| 22 | Albumin | Rejected | 0 | 0 | 100 |
| 23 | All vasoactive agents | Rejected | 0 | 0 | 100 |
| 24 | MI | Rejected | 0 | 0 | 100 |
| 25 | ARB | Rejected | 0 | 0 | 100 |
| 26 | Routine CABG | Rejected | 0 | 0 | 100 |
| 27 | Cardiomyopathy | Rejected | 0 | 0 | 100 |
| 28 | Cerebrovascular disease | Rejected | 0 | 0 | 100 |
| 29 | CKD | Rejected | 0 | 0 | 100 |
| 30 | COPD | Rejected | 0 | 0 | 100 |
| 31 | Diabetes | Rejected | 0 | 0 | 100 |
| 32 | Diuretics | Rejected | 0 | 0 | 100 |
| 33 | Emergency CABG | Rejected | 0 | 0 | 100 |
| 34 | Hematocrit | Rejected | 0 | 0 | 100 |
| 35 | Heart Failure | Rejected | 0 | 0 | 100 |
| 36 | Hemoglobin | Rejected | 0 | 0 | 100 |
| 37 | Hyperlipidemia | Rejected | 0 | 0 | 100 |
| 38 | Hypertension | Rejected | 0 | 0 | 100 |
| 39 | Malignancy | Rejected | 0 | 0 | 100 |
| 40 | Neutrophils | Rejected | 0 | 0 | 100 |
| 41 | Platelet count | Rejected | 0 | 0 | 100 |
| 42 | Potassium | Rejected | 0 | 0 | 100 |
| 43 | Sex | Rejected | 0 | 0 | 100 |
| 44 | PCA | Rejected | 0 | 0 | 100 |
| 45 | Mechanical Ventilation | Rejected | 0 | 0 | 100 |
