# Supplementary material for: Blood Count-Based Inflammatory Indices Do Not Improve Prediction of Acute Kidney Injury in Cardiogenic Shock
Source: J Clin Med Res. 2026 Aug 26;18(8):528–41. doi: 10.14740/jocmr6666 (PMC13568768; doi:10.14740/jocmr6666)
Supplement: Supplementary file 3 [file jocmr-18-08-528-s003.docx]

**Suppl 3.** Boruta variable-importance distributions for acute kidney injury classification. Boxplots summarize variable-importance scores across Boruta iterations. Green indicates confirmed variables, red indicates rejected variables, and blue indicates shadow features used as reference comparators.
