# Supplementary material for: Blood Count-Based Inflammatory Indices Do Not Improve Prediction of Acute Kidney Injury in Cardiogenic Shock
Source: J Clin Med Res. 2026 Aug 26;18(8):528–41. doi: 10.14740/jocmr6666 (PMC13568768; doi:10.14740/jocmr6666)
Supplement: Supplementary file 4 [file jocmr-18-08-528-s004.docx]

**Suppl 4.** Exploratory Elastic-net Model Performance Across MICE-Imputed Datasets

| **Model** | **Lambda min** | **Lambda 1se** | **Mean Apparent AUC** |
| --- | --- | --- | --- |
| Elastic-net logistic regression, alpha = 0.5 | 0.14 | 0.20 | 0.69 |

AUC represents apparent discrimination from the exploratory elastic-net model. Elastic-net analyses were used to evaluate variable-selection stability and were not used as the primary inferential model. *Dropped because of low elastic net frequency.
