# Supplementary material for: Blood Count-Based Inflammatory Indices Do Not Improve Prediction of Acute Kidney Injury in Cardiogenic Shock
Source: J Clin Med Res. 2026 Aug 26;18(8):528–41. doi: 10.14740/jocmr6666 (PMC13568768; doi:10.14740/jocmr6666)
Supplement: Supplementary file 5 [file jocmr-18-08-528-s005.docx]

**Suppl 5.** Exploratory Elastic-Net Variable Selection Frequency Across MICE-Imputed Datasets

| **Predictor** | **Missing n (%)** | **Boruta decision** | **Boruta confirmed (%)** | **Selection frequency across imputed datasets** | **Coef mean (pooled)** | **Coef SE (pooled)** | **OR (95%)** | **Df (Barnard, Rubin** | **P (pooled, Rubin df)** |
| --- | --- | --- | --- | --- | --- | --- | --- | --- | --- |
| BUN | 28 (6.7%) | Confirmed | 100 | 1 | 0.01 | 0.01 | 1.01 (0.99-1.02) | 276.63 | 0.34 |
| Creatinine | 28 (6.7%) | Confirmed | 100 | 1 | 0.60 | 0.18 | 1.83 (1.28-2.60) | 222.28 | 0.001 |
| ALT | 97 (23.2%) | Confirmed | 100 | 0.95 | 0.00 | 0.00 | 1.0 (1.0-1.001) | 166.88 | 0.02 |
| AST | 98 (23.4%) | Confirmed | 100 | 0.05 | * | * | * | * | * |
| Lactate/albumin | 143 (34.1%) | Confirmed | 100 | 0.05 | * | * | * | * | * |
