# Supplementary material for: Blood Count-Based Inflammatory Indices Do Not Improve Prediction of Acute Kidney Injury in Cardiogenic Shock
Source: J Clin Med Res. 2026 Aug 26;18(8):528–41. doi: 10.14740/jocmr6666 (PMC13568768; doi:10.14740/jocmr6666)
Supplement: Supplementary file 6 [file jocmr-18-08-528-s006.docx]

**Suppl 6.** MICE imputed pooled multivariable logistic regression analysis excluding creatinine and BUN

| **Variable** | **Adjusted OR** | **95% CI** |  | **P value** |
| --- | --- | --- | --- | --- |
| Age | 0.93 | 0.75 | 1.16 | 0.53 |
| Male sex | 1.07 | 0.685 | 1.68 | 0.76 |
| SOFA score | 1.09 | 0.86 | 1.38 | 0.47 |
| Chronic renal disease | 1.84 | 1.06 | 3.20 | 0.03 |
| ALT | 1.56 | 0.63 | 3.85 | 0.33 |
| AST | 0.96 | 0.35 | 2.58 | 0.93 |
| SII | 1.22 | 0.54 | 2.79 | 0.63 |
| AISI | 0.69 | 0.28 | 1.68 | 0.41 |
| NLR | 0.76 | 0.32 | 1.83 | 0.55 |
| PLR | 1.22 | 0.74 | 2.01 | 0.44 |
| MLR | 1.13 | 0.71 | 1.80 | 0.61 |
| SIRI | 1.26 | 0.45 | 3.52 | 0.67 |
| NPAR | 1.10 | 0.82 | 1.38 | 0.63 |
| Lactate-to-albumin ratio | 1.14 | 0.74 | 1.73 | 0.55 |

In this sensitivity analysis, continuous variables were standardized and creatinine and blood urea nitrogen were excluded. Odds ratios (OR) represent the change in odds of AKI per 1-standard deviation increase in the predictor. Logistic regression estimates were pooled across 20 multiply imputed datasets.
