# Supplementary material for: Blood Count-Based Inflammatory Indices Do Not Improve Prediction of Acute Kidney Injury in Cardiogenic Shock
Source: J Clin Med Res. 2026 Aug 26;18(8):528–41. doi: 10.14740/jocmr6666 (PMC13568768; doi:10.14740/jocmr6666)
Supplement: Supplementary file 7 [file jocmr-18-08-528-s007.docx]

**Suppl 7.** Sensitivity Analysis of Inflammatory Markers for AKI discrimination After Excluding Renal Function Markers

|  | **Non renal clinical model** | **Non renal clinical model plus Inflammatory markers** | **Delta AUC** |
| --- | --- | --- | --- |
| Apparent AUC | 0.62 (0.55 to 0.66) | 0.63 (0.56 to 0.65) | 0.002 (-0.06 to 0.08) |
| Out-of-fold AUC | 0.52 (0.49 to 0.56) | 0.52 (0.47 to 0.56) | -0.004 (-0.07 to 0.04) |

In the sensitivity analysis which excluded creatinine and blood nitrogen the addition of inflammatory markers demonstrated no improvement in discrimination.
